# High-temperature operation of electrical injection type-II (GaIn)As/Ga(AsSb)/(GaIn)As “W”-quantum well lasers emitting at 1.3 µm

**DOI:** 10.1038/s41598-018-19189-1

**Published:** 2018-01-23

**Authors:** C. Fuchs, A. Brüggemann, M. J. Weseloh, C. Berger, C. Möller, S. Reinhard, J. Hader, J. V. Moloney, A. Bäumner, S. W. Koch, W. Stolz

**Affiliations:** 1 Materials Sciences Center and Department of Physics, Philipps-Universität Marburg, Renthof 5, 35032 Marburg, Germany; 2 Nonlinear Control Strategies Inc., 7040 N. Montecatina Dr., Tucson, AZ 85704 USA; 3 College of Optical Sciences, University of Arizona, 1630 E. University Blvd., Tucson, AZ 85721 USA

## Abstract

Electrical injection lasers emitting in the 1.3 μm wavelength regime based on (GaIn)As/Ga(AsSb)/(GaIn)As type-II double “W”-quantum well heterostructures grown on GaAs substrate are demonstrated. The structure is designed by applying a fully microscopic theory and fabricated using metal organic vapor phase epitaxy. Temperature-dependent electroluminescence measurements as well as broad-area edge-emitting laser studies are carried out in order to characterize the resulting devices. Laser emission based on the fundamental type-II transition is demonstrated for a 975 μm long laser bar in the temperature range between 10 °C and 100 °C. The device exhibits a differential efficiency of 41 % and a threshold current density of 1.0 kA/cm^2^ at room temperature. Temperature-dependent laser studies reveal characteristic temperatures of T_0_ = (132 ± 3) K over the whole temperature range and T_1_ = (159 ± 13) K between 10 °C and 70 °C and T_1_ = (40 ± 1) K between 80 °C and 100 °C.

## Introduction

The development of efficient near-infrared lasers emitting at a wavelength of 1.3 μm is of great interest due to their application in optical telecommunications based on the O-band (1.26 µm to 1.36 µm). Present-day telecommunication systems typically use InP-based materials systems such as (GaIn)(AsP)/InP^1^ or (AlGaIn)As/InP^2–5^. While these materials have proven to be a spectrally suitable choice for telecommunication systems, their performance is considerably affected by Auger losses as well as potentially small hetero band offsets. A possible solution to these problems is the application of GaAs-based lasers which typically offer large hetero band offsets and mature (AlGa)As/GaAs-based technology. These improvements can prevent carrier leakage and facilitate the fabrication of sophisticated device structures such as vertical-cavity surface-emitting lasers (VCSELs). However, Auger recombination has to be considered as a loss mechanism which is primarily defined by the electronic structure of the active material. While the application of (GaIn)(NAs)/GaAs^6–8^ or Ga(AsSb)/GaAs^9–11^ as active materials are interesting due to their spectral properties, these materials systems do not provide a distinct route towards the suppression of Auger losses.

Type-II heterostructures were suggested as a possible solution to this problem^12,13^. The conduction and valence band states are dominated by different materials in these heterostructures and thus, it is possible to independently modify these energy levels which allows for a more flexible band structure engineering and offers the opportunity to tune the Auger coefficients. The successful demonstration of type-II emission based on metal organic vapor phase epitaxy (MOVPE)-grown (GaIn)As/Ga(AsSb) bilayer quantum wells (QWs)^14^ was followed by various attempts to fabricate lasers based on these materials. The first demonstration of an electrical injection laser based on a bilayer structure was reported by Klem *et al*.^15^. The device exhibited a differential efficiency of 38 % and a maximum optical output pulse power of 0.14 W per facet at a wavelength of 1.17 µm. The first attempt to improve the performance of these devices was reported by Ryu and Dapkus in 2002 by applying “W”-QW heterostructures (“W”-QWH) in order to improve the wave function overlap of the spatially separated electrons and holes^16^.

In our previous studies, we applied MOVPE in order to fabricate (GaIn)As/Ga(AsSb)/(GaIn)As “W”-QWHs for near-infrared laser applications. A comparison of experimental and theoretical photoluminescence spectra obtained from a fully microscopic theory yielded an excellent agreement. Furthermore, material gain values similar to those of type-I heterostructures were predicted^17^. In order to confirm the results obtained from theoretical modeling and to analyze the energy states that are involved in the optical transitions in these “W”-QWHs, photomodulated reflectance spectroscopy was employed. It was possible to confirm the existence of two bound electron states as well as up to three bound hole states depending on the antimony concentration^18^. These novel insights were used to fabricate vertical-external-cavity surface-emitting lasers (VECSELs) operating in the 1.2 µm wavelength regime. A continuous wave output power of 4 W was achieved under multi mode operating conditions^19^. Furthermore, an output power of 0.35 W with a beam quality factor below 1.2 under transverse single mode operating conditions was observed at room temperature^20^. Low threshold electrical injection lasers emitting in the 1.2 µm regime with differential efficiencies of 66 % and a maximum optical output pulse power of 1.4 W per facet were demonstrated using broad-area edge-emitting lasers under pulsed excitation conditions^21^. Additionally, a MOVPE growth study demonstrated the possibility to adapt the design of the active “W”-QWH for emission at 1.3 µm by increasing the antimony concentration to approximately 27 %^22^.

In the present publication, a fully microscopic theory is applied to optimize the (GaIn)As/Ga(AsSb)/(GaIn)As “W”-QWH for an application as active region in lasers emitting at 1.3 µm. The structure proposed by this design study is fabricated using MOVPE and characterized by spectral measurements below and above laser threshold as well as measurements of laser characteristics in the temperature range between 10 °C and 100 °C.

## Results

### Theoretical design of the “W”-QWH active region

The “W”-QWH is designed using the fully microscopic theory described by Berger *et al*. in Ref.^17^, which was previously applied to various type-I^23^ as well as type-II^24^ materials systems. Only nominal material parameters such as QW thicknesses and compositions are required as input for this approach. The systems used in our previous laser studies consist of 6 nm thick electron and 4 nm thick hole quantum wells containing 20 % In and Sb, respectively, resulting in an emission wavelength of 1.2 µm. This design yields an overall thickness of the electron confinement potential of 16 nm. While the fabrication of these structures is possible, their application is challenging due to higher order type-II transitions that are predicted to dominate the material gain spectra at high charge carrier densities^25^. This behavior results from the electron energy states being nearly degenerate due to the large thickness of the electron confinement potential^18^.

The adaption of the active region for applications at 1.3 µm using the fully microscopic theory yields concentrations of 22 % In and 28 % Sb while maintaining layer thicknesses of 6 nm and 4 nm, respectively. Furthermore, the microscopic theory is applied to calculate the indium concentration that is required to retain a constant emission wavelength while decreasing the (GaIn)As QW thicknesses in order to increase the energetic separation between the electron ground state and the first excited electron state. A thickness of 4 nm is chosen since the increase in quantization energy requires an experimentally accessible indium concentration of 28 %. The Ga(AsSb) QW remains unchanged with an antimony concentration of 28 % and a thickness of 4 nm. Altogether, these changes result in a low excitation density photoluminescence peak at a wavelength of 1.34 µm for a carrier density of 0.002 × 10^12 ^/cm^2^, a material gain value of 750/cm at a wavelength of 1.31 µm and a separation of the electron ground state and the first excited electron state of 22 meV for a carrier density of 3.0 × 10^12^/cm^2^. A comparison with the reference structure consisting of 6 nm thick (GaIn)As QWs containing 22 % indium and the same Ga(AsSb) QW yields improvements of the energetic separation of the electron ground state and the first excited electron state of 38 % as well as of the material gain value of 66 % at the above mentioned carrier density. Additionally, the strain thickness product per “W”-QWH is decreased by 11 % allowing for a more flexible device design in highly strained devices such as VECSELs^19^.

### Temperature-dependent spectral properties

**Figure 1:**
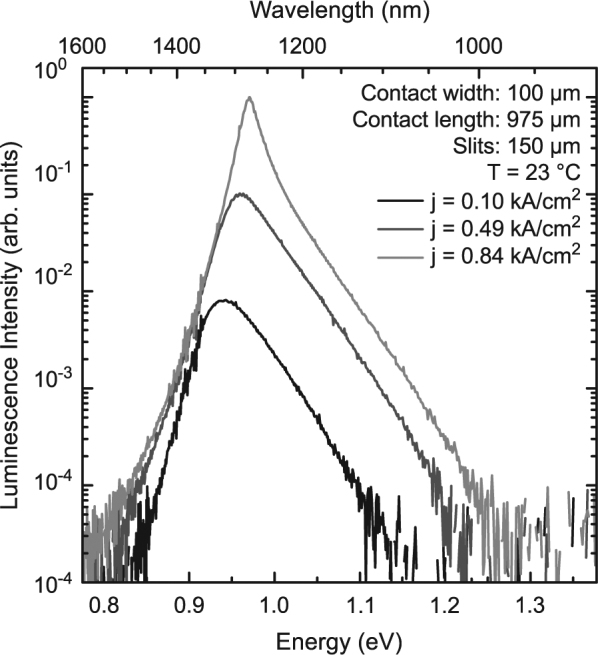
EL spectra of a 975 μm long (GaIn)As/Ga(AsSb)/(GaIn)As double “W”-QWH laser at a temperature of 23 °C. All spectra shown here are recorded below laser threshold for current densities between 0.10 kA/cm^2^ and 0.84 kA/cm^2^. The spectra are normalized with respect to the 0.84 kA/cm^2^ measurement and monochromator slit widths of 150 µm are used for all measurements.

Room temperature electroluminescence (EL) spectra of a 975 µm long (GaIn)As/Ga(AsSb)/(GaIn)As double “W”-QWH laser are shown in Fig. 1. The EL peak wavelength shifts from 1.320 µm at a current density of 0.1 kA/cm^2^ to 1.278 µm at 0.84 kA/cm^2^. A linear fit yields a blue shift of (42 ± 7) meV/(kA/cm^2^) which was also observed in previous laser studies^21^ and thoroughly analyzed using microscopic models^17,25,26^. The mode narrowing starting from 0.84 kA/cm^2^ results in laser operation at 1.275 µm. Thus, the double “W”-QWH design used for this device efficiently suppresses higher order type-II transitions by establishing suitable charge carrier densities per “W”-QWH.

**Figure 2:**
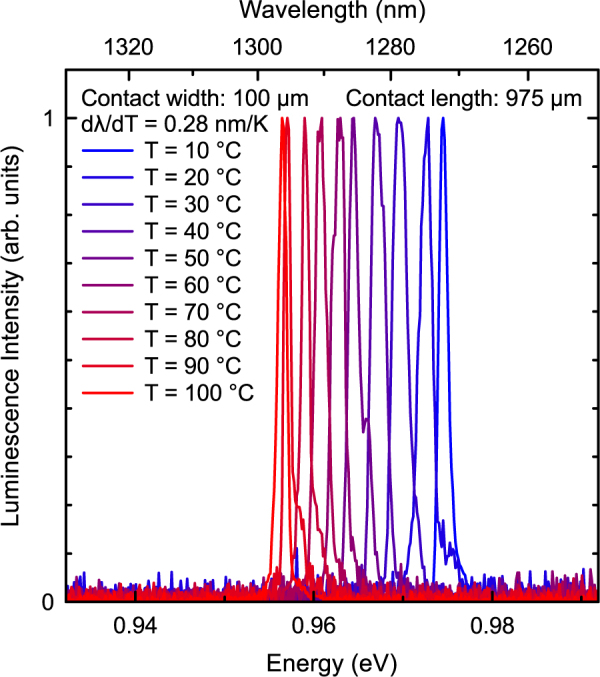
Normalized laser spectra directly above laser threshold of a 975 µm long (GaIn)As/Ga(AsSb)/(GaIn)As double “W”-QWH laser for temperatures between 10 °C and 100 °C. A linear fit of the peak wavelengths yields a shift rate of 0.28 nm/K.

Temperature-dependent spectral laser measurements directly above laser threshold are carried out in order to verify that the laser emission is based on the fundamental type-II transition up to a temperature of 100 °C. Therefore, the emission directly above laser threshold is analyzed for temperatures between 10 °C and 100 °C. The resulting spectra are presented in Fig. 2. A temperature-induced average shift rate of 0.28 nm/K is observed corresponding to a shift of the emission wavelength from 1.272 µm (10 °C) to 1.296 µm (100 °C). However, no switching to higher order type-II transitions is observed and thus, stable laser operation based on the fundamental type-II transition up to a temperature of 100 °C is obtained using the present design.

### Temperature-dependent laser studies

**Figure 3:**
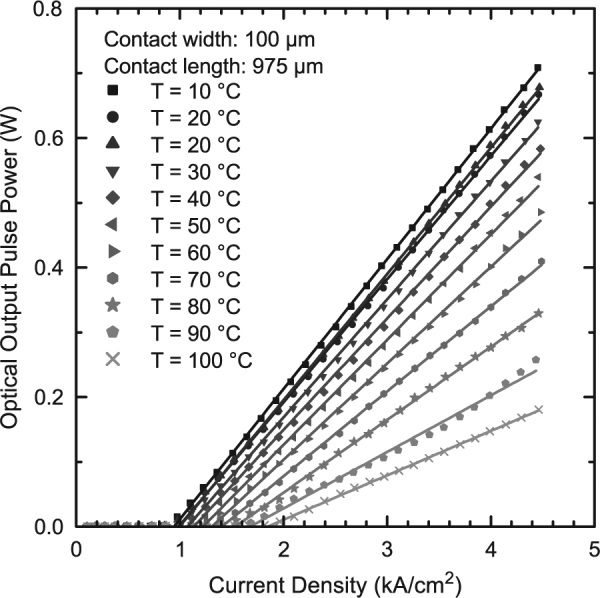
Laser characteristics of a 975 µm long (GaIn)As/Ga(AsSb)/(GaIn)As double “W”-QWH laser at temperatures between 10 °C and 100 °C. All optical output pulse power values presented in this graph are single facet values. Laser emission is based on the fundamental type-II transition up to a setup-limited temperature of 100 °C.

The knowledge of the emission wavelengths enables the measurement of laser characteristics using an appropriate detector calibration for each temperature. Laser characteristics of a 975 µm long (GaIn)As/Ga(AsSb)/(GaIn)As double “W”-QWH laser are shown in Fig. 3 for temperatures between 10 °C and 100 °C. The room temperature measurement (20 °C) is carried out before and after the temperature series in order to verify that no device degradation occurs during the temperature series. A differential efficiency of 41 %, a threshold current density of 1.0 kA/cm^2^, and a maximum optical output pulse power of 0.68 W per facet at a power supply-limited current density of 4.5 kA/cm^2^ are observed at a wavelength of 1.275 µm. Furthermore, simple cavity length dependent studies of the present laser structure yield internal losses of (0.7 ± 1.6)/cm and an internal efficiency of (37.1 ± 6.4)% at a temperature of 20 °C. While this low internal loss value is excellent, additional experiments are underway in order to analyze whether cavity length dependent studies are an appropriate tool for the characterization of internal losses in type-II lasers^1,4,5^. A possible reason for deviations may be the fact that the confinement potentials are distorted as charge carriers are filled into the active region resulting in a strong carrier density dependence of the wave functions. Thus, the internal efficiency as well as the internal losses might strongly depend on the actual carrier density in the type-II active region. Laser operation is obtained up to a setup-limited temperature of 100 °C and an output power of 0.18 W is achieved at this temperature. Thus, the present device concept can be utilized under the typical operating conditions found in telecommunication laser applications.

**Figure 4:**
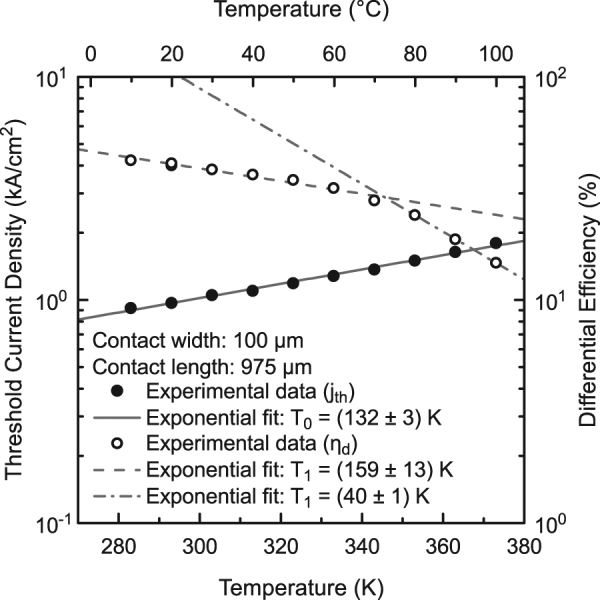
Temperature dependence of the of the threshold current density and differential efficiency of the of a 975 µm long (GaIn)As/Ga(AsSb)/(GaIn)As double “W”-QWH laser. Exponential fits yield characteristic temperatures of T_0_ = (132 ± 3) K over the whole temperature range and T_1_ = (159 ± 13) K between 10 °C and 70 °C and T_1_ = (40 ± 1) between 80 °C and 100 °C.

A plot of the threshold current density and the differential efficiency in combination with exponential fits reveals characteristic temperatures of T_0_ = (132 ± 3) K over the whole temperature range and T_1_ = (159 ± 13) K between 10 °C and 70 °C and T_1_ = (40 ± 1) K between 80 °C and 100 °C as shown in Fig. 4. These promising values for T_0_ and T_1_ up to a temperature of 70 °C once again underline the potential of type-II heterostructures. Additional experiments are underway to also quantify Auger losses in these type-II “W”-QWH lasers and to determine the origin of the decrease of T_1_ above 70 °C. A comparison with literature values of InP-based materials systems is difficult since the actual device design as well as facet coatings have an influence on the characteristic temperatures^3^. Typical T_0_ values reported in the literature are T_0_ = 50K – 80K and T_0_ = 100K – 120K for (GaIn)(AsP)/InP^1^ and (AlGaIn)As/InP^2–5^, respectively, in case of ridge waveguide and buried-heterostructure lasers.

## Summary and Conclusion

In conclusion, the careful optimization of (GaIn)As/Ga(AsSb)/(GaIn)As “W”-QWHs for emission at 1.3 μm using a fully microscopic theory resulted in an improvement of the material gain values by 66 % compared to previous designs. The suggested structures were fabricated using MOVPE and their characterization yielded room temperature laser operation based on the fundamental type-II transition at 1.275 µm. Furthermore, a differential efficiency of 41 %, a threshold current density of 1.0 kA/cm^2^, and a maximum optical output pulse power of 0.68 W per facet at a power supply-limited current density of 4.5 kA/cm^2^ were obtained from characteristics in case of a 975 µm long laser bar at room temperature. The temperature-dependent characterization of the device yielded operation based on the fundamental type-II transition up to a temperature of 100 °C as well as characteristic temperatures of T_0_ = (132 ± 3) K over the whole temperature range and T_1_ = (159 ± 13) K between 10 °C and 70 °C and T_1_ = (40 ± 1) K between 80 °C and 100 °C. These results indicate that the (GaIn)As/Ga(AsSb)/(GaIn)As materials system grown on GaAs substrate is a promising candidate for efficient and temperature-stable telecommunication lasers emitting at 1.3 µm. Further research should be carried out in order to improve the active region as well as the device design and the growth conditions of the active region in order to achieve a reduction of the threshold current density. The excellent threshold current densities obtained using (GaIn)(NAs)/GaAs active regions, which are considerably smaller than 1.0 kA/cm^2^^7,8^, should serve as a benchmark during these investigations. Additionally, the suppression of Auger losses needs to be confirmed and advanced device concepts such as VCSELs and distributed feedback lasers based on this materials system should be demonstrated and investigated.

## Experimental Methods

The epitaxial growth of the sample is carried out in an AIXTRON AIX 200 GFR (Gas Foil Rotation) reactor system using H_2_ as carrier gas at a reactor pressure of 50 mbar. Triethylgallium (TEGa), trimethylindium (TMIn), and trimethylaluminum (TMAl) are applied as group-III, tertiarybutylarsine (TBAs) and triethylantimony (TESb) are applied as group-V, and diethyltellurium (DETe) and tetrabromomethane (CBr_4_) are applied as dopant sources, respectively. The n-GaAs (001) (± 0.1°) substrates are treated with a TBAs-stabilized bake-out procedure in order to remove the native oxide layer before depositing a tellurium-doped GaAs buffer as well as the laser structure. The laser structure consists of 1.4 µm thick (Al_0.4_Ga_0.6_)As cladding layers, 0.2 µm thick GaAs separate confinement heterostructures (SCHs), and two active “W”-QWHs. Furthermore, a 20 nm thick GaAs intermediate barrier is grown in between the “W”-QWHs in order to ensure quantum mechanically separated systems. A highly carbon-doped GaAs cap is added on top of the p-(AlGa)As cladding in order to ensure small contact resistances. This layer is doped using CBr_4_ as dopant sources while the p-(AlGa)As cladding is carbon-doped by employing a decreased V/III ratio. The growth of the n-GaAs buffer, the n- and p-(AlGa)As claddings, the SCHs, and the p^+^-GaAs cap is carried out at a growth temperature of 625 °C, while the growth temperature is lowered to 550 °C for the growth of the active regions as well as for the GaAs intermediate barrier. The resulting double “W”-QWH laser structure is processed as outlined in Ref.^21^. in order to obtain broad-area edge-emitting lasers with cavity lengths between 750 µm and 1990 µm.

The detailed device characterization is carried out under pulsed excitation conditions using a pulse length of 400 ns at a repetition rate of 10 kHz. Laser characteristics are measured using a large-area germanium photodetector for the detection of the single-facet output power as a function of the injection current density. EL measurements below laser threshold are performed using a custom-built setup in which the optical signal is dispersed by a grating monochromator (Jobin-Yvon THR 1000) and detected using a liquid nitrogen cooled germanium detector in combination with a lock-in amplifier (Stanford Research Systems SR510). EL measurements above laser threshold are carried out using an optical spectrum analyzer (Yokogawa AQ6370B). All EL measurements are carried out in a p-side up geometry and the temperature is adjusted using a thermoelectric heater.
