# Author Correction: High-temperature operation of electrical injection type-II (GaIn)As/Ga(AsSb)/(GaIn)As “W”-quantum well lasers emitting at 1.3 µm

**DOI:** 10.1038/s41598-018-25808-8

**Published:** 2018-05-15

**Authors:** C. Fuchs, A. Brüggemann, M. J. Weseloh, C. Berger, C. Möller, S. Reinhard, J. Hader, J. V. Moloney, A. Bäumner, S. W. Koch, W. Stolz

**Affiliations:** 1 Materials Sciences Center and Department of Physics, Philipps-Universität Marburg, Renthof 5, 35032 Marburg, Germany; 2 Nonlinear Control Strategies Inc., 7040 N. Montecatina Dr., Tucson, AZ 85704 USA; 3 College of Optical Sciences, University of Arizona, 1630 E. University Blvd., Tucson, AZ 85721 USA

Correction to: *Scientific Reports* 10.1038/s41598-018-19189-1, published online 23 January 2018

In Figure 1, there is a typographical error in the y-axis of the scale bar. The correct Figure 1 appears below.

**Figure 1:**
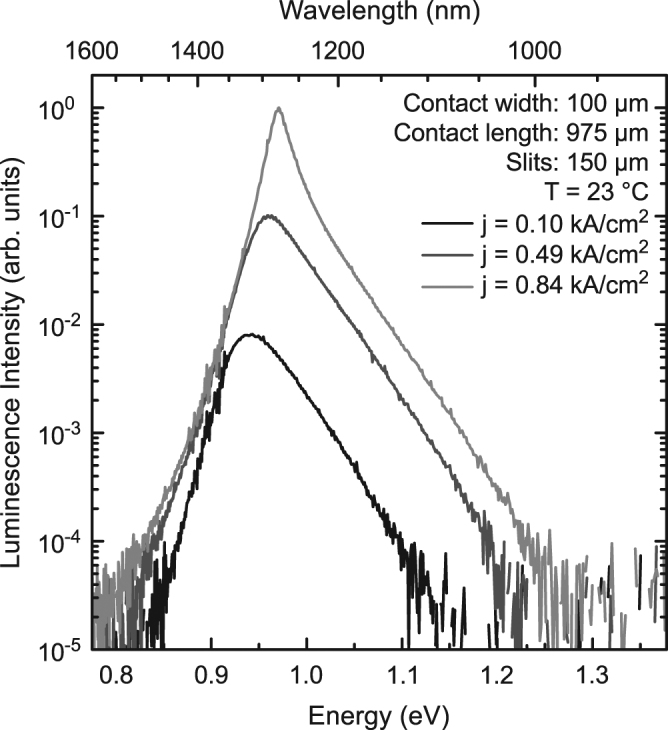
EL spectra of a 975 μm long (GaIn)As/Ga(AsSb)/(GaIn)As double “W”-QWH laser at a temperature of 23 °C. All spectra shown here are recorded below laser threshold for current densities between 0.10 kA/ cm^2^ and 0.84 kA/cm^2^. The spectra are normalized with respect to the 0.84 kA/cm^2^ measurement and monochromator slit widths of 150 μm are used for all measurements.

